# *N*-[(2-Chloro-3-quinol­yl)meth­yl]-4-fluoro­aniline

**DOI:** 10.1107/S1600536810036056

**Published:** 2010-09-11

**Authors:** Jerry P. Jasinski, Albert E. Pek, C. S. Chidan Kumar, H. S. Yathirajan, Suresh Kumar

**Affiliations:** a Department of Chemistry, Keene State College, 229 Main Street, Keene, NH 03435-2001, USA; b Department of Studies in Chemistry, University of Mysore, Manasagangotri, Mysore 570 006, India; c Department of Pharmaceutical Chemistry, Faculty of Pharmacy, Hamdard University, Jamia Hamdard, New Delhi 110 062, India

## Abstract

In the title compound, C_16_H_12_ClFN_2_, the dihedral angle between the quinoline ring system and the flourophenyl ring is 86.70 (4)°. In the crystal, mol­ecules are linked into chains along the *a* axis by N—H⋯N hydrogen bonds. In addition, C—H⋯π inter­actions involving the two benzene rings are observed.

## Related literature

For general background, properties and the biological activity of quinolines, see: Campbell *et al.* (1988 ▶); Dutta *et al.* (2002 ▶); Markees *et al.* (1970 ▶); Meth-Cohn *et al.* (1981 ▶); Michael *et al.* (1997 ▶); Morimoto *et al.* (1991 ▶); Padwa *et al.* (1999 ▶); Rajendran & Karavembu (2002 ▶); Robert & Meunier *et al.* (1998 ▶). For the synthesis of quinolines, see: Kouznetsov *et al.* (2005 ▶). For related structures, see: Butcher *et al.* 2007 ▶); Lynch *et al.* (2001 ▶); Subashini *et al.* (2009 ▶); Yathirajan *et al.* (2007 ▶); Wu *et al.* (2009 ▶); Khan *et al.* (2010 ▶). For bond-length data, see: Allen *et al.* (1987 ▶) .
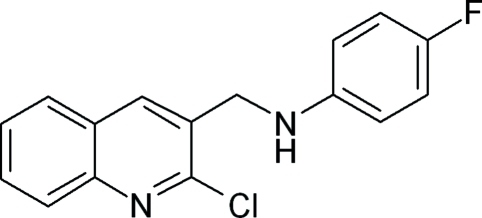


## Experimental

### 

#### Crystal data

C_16_H_12_ClFN_2_
*M* *_r_* = 286.73
Triclinic, 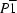

*a* = 7.3661 (8) Å
*b* = 8.8967 (9) Å
*c* = 11.5129 (12) Å
α = 68.704 (1)°
β = 74.468 (1)°
γ = 75.445 (1)°
*V* = 667.25 (12) Å^3^
*Z* = 2
Mo *K*α radiation
μ = 0.29 mm^−1^
*T* = 100 K
0.55 × 0.50 × 0.25 mm

#### Data collection

Bruker APEXII CCD area-detector diffractometer
Absorption correction: multi-scan (*SADABS*; Bruker, 2008 ▶) *T*_min_ = 0.858, *T*_max_ = 0.932
8162 measured reflections
3930 independent reflections
3633 reflections with *I* > 2σ(*I*)
*R*_int_ = 0.014

#### Refinement

*R*[*F* ^2^ > 2σ(*F* ^2^)] = 0.034
*wR*(*F* ^2^) = 0.094
*S* = 1.03
3930 reflections
181 parameters
H-atom parameters constrained
Δρ_max_ = 0.49 e Å^−3^
Δρ_min_ = −0.30 e Å^−3^

### 

Data collection: *APEX2* (Bruker, 2008 ▶); cell refinement: *SAINT* (Bruker, 2008 ▶); data reduction: *SAINT*; program(s) used to solve structure: *SHELXTL* (Sheldrick, 2008 ▶); program(s) used to refine structure: *SHELXTL*; molecular graphics: *SHELXTL*; software used to prepare material for publication: *SHELXTL* and *PLATON* (Spek, 2009 ▶).

## Supplementary Material

Crystal structure: contains datablocks global, I. DOI: 10.1107/S1600536810036056/ci5158sup1.cif

Structure factors: contains datablocks I. DOI: 10.1107/S1600536810036056/ci5158Isup2.hkl

Additional supplementary materials:  crystallographic information; 3D view; checkCIF report

## Figures and Tables

**Table 1:** Hydrogen-bond geometry (Å, °) *Cg*1 and *Cg*2 are the centroids of the C1–C6 and C11–C16 rings, respectively.

*D*—H⋯*A*	*D*—H	H⋯*A*	*D*⋯*A*	*D*—H⋯*A*
N2—H18⋯N1^i^	0.86	2.30	3.1353 (12)	165
C4—H4⋯*Cg*2^ii^	0.93	2.91	3.7494 (13)	151
C10—H10*A*⋯*Cg*1^iii^	0.97	2.62	3.5365 (12)	157
C10—H10*B*⋯*Cg*2^iv^	0.97	2.98	3.8083 (11)	145

Symmetry codes: (i) 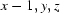; (ii) 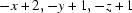; (iii) 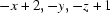; (iv) 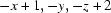.

**Fig. 1.**
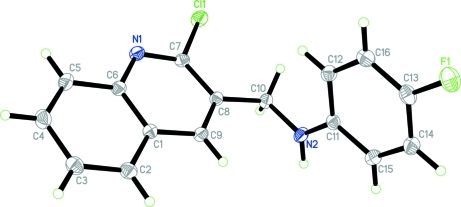
Molecular structure of the title compound, showing the atom-labeling scheme and 50% probability displacement ellipsoids.

**Fig. 2.**
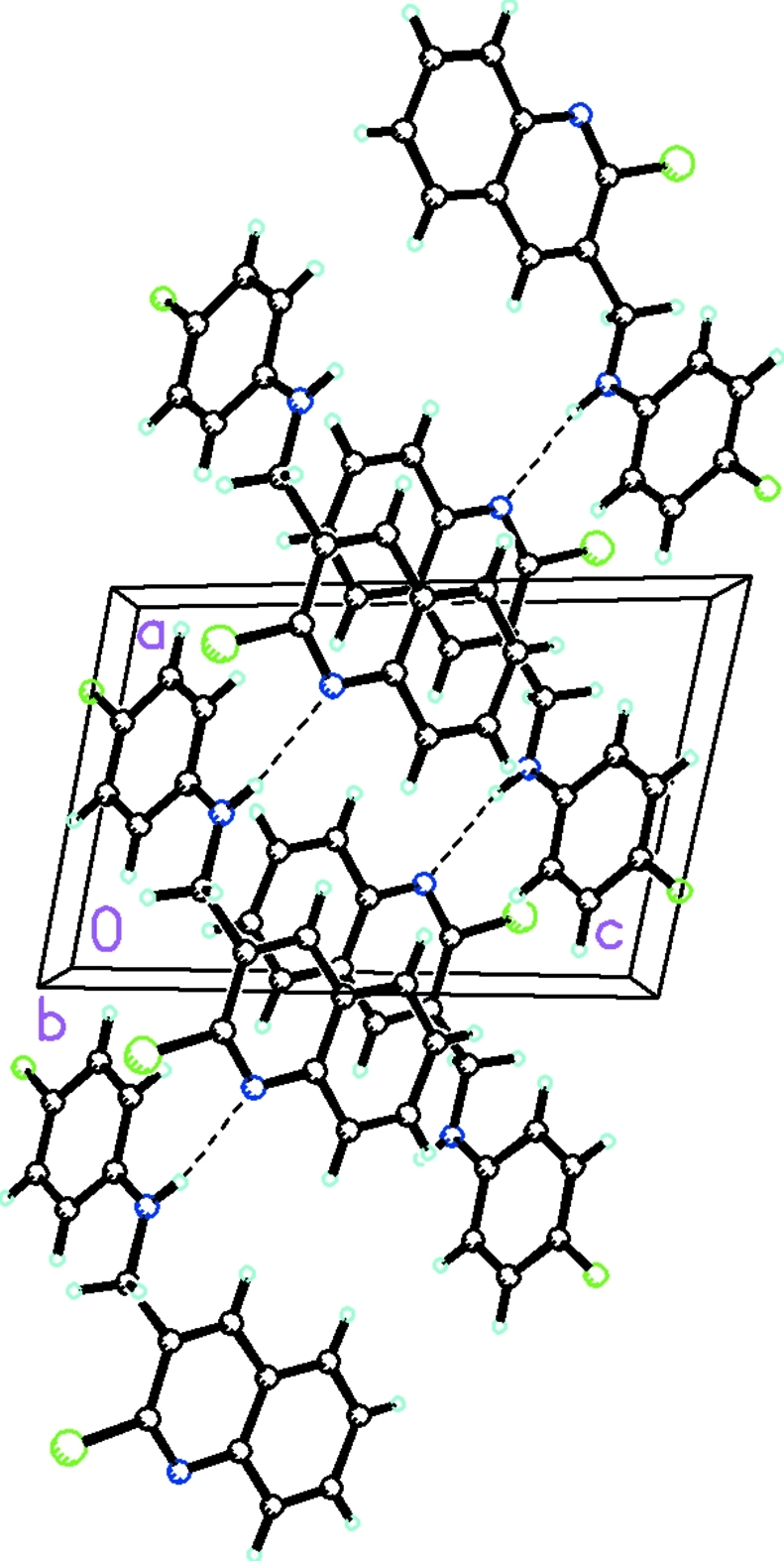
Packing diagram of the title compound, viewed down the b axis. Dashed lines indicate weak N—H···N hydrogen bonds.

**Fig. 3.**
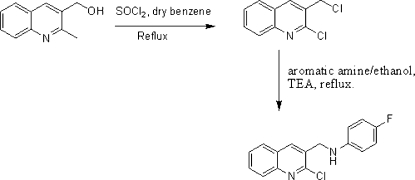
Reaction scheme for the title compound.

## supplementary crystallographic information

### Comment

Quinoline derivatives represent a major class of heterocycles, and a number of preparations using them have been known since the late 1800s. Quinolines are found in natural products (Morimoto *et al.*, 1991; Michael *et al.*, 1997), numerous commercial products, including fragrances, dyes (Padwa *et al.*, 1999) and biologically active compounds (Markees *et al.*, 1970; Campbell *et al.*, 1988). Quinoline alkaloids such as quinine, chloroquin, mefloquine and amodiaquine are used as efficient drugs for the treatment of malaria (Robert & Meunier, 1998). Several quinoline derivatives have been evaluated *in vitro* against a number of parasites of HTLV-1 transformed cells. 2-Chloro substituted quinolines are vital synthetic intermediates in the construction of a large number of linearly fused tri- and tetra- cyclic quinolines studied for the DNA intercalating properties (Meth-Cohn *et al.*, 1981; Rajendran & Karavembu, 2002; Dutta *et al.*, 2002). A review on recent progress in the synthesis of quinolines (Kouznetsov *et al.* 2005) has been described. The crystal structure studies of 8-chloro-2-methylquinoline (Wu *et al.*, 2009), 2-chloro-4-methylquinoline (Lynch *et al.*, 2001), 4-chloro-8-(trifluoromethyl)quinoline (Yathirajan *et al.*, 2007), 1-(quinolin-2-yl)ethanone (Butcher *et al.*, 2007) and 2-chloro-7-methylquinoline-3-carbaldehyde (Subashini *et al.*, 2009) have been reported. In view of the importance of quinolines, the paper reports the synthesis and crystal structure of the title compound.

In the title molecule (Fig. 1), the 2-chloroquinoline ring system and 4-fluoroaniline ring are bonded to a methane carbon, C10. The dihedral angle between the mean planes of the planar chloroquinoline ring system (dihedral angle between rings = 0.92 (5)°) and the flourophenyl ring is 86.70 (4)°. Bond distances (Allen *et al.*, 1987) and angles are in normal ranges.

The molecules are linked into chains along the *a* axis by N—H···N hydrogen bonds (Fig. 2). In addition, C—H···π interactions involving the two benzene rings (Table 1) influence crystal packing in the unit cell.

### Experimental

In a mixture of 3-(chloromethyl)-2-chloroquinoline (0.003 mol) and substituted phenyl amine (0.003 mol) in 20 ml of absolute ethanol, 1 ml of triethylamine (TEA) was added and refluxed for 12–15 hrs (Fig. 3). After completion of the reaction, content of the flask was reduced to half and left overnight. The crystalline mass obtained was filtered off, washed with water, dried and re-crystallized from ethanol to give *N*-[(2-chloroquinolin-3-yl)methyl]-4-fluoroaniline. X-ray quality crystals were obtained by slow evaporation of a methanol solution (m.p. 413–415 K).

### Refinement

All of the H atoms were placed in their calculated positions and then refined using the riding model with atom—H lengths of 0.93 Å (CH), 0.97 Å (CH_2_) or 0.86 Å (NH). Isotropic displacement parameters for these atoms were set to 1.2 times *U*_eq_ of the parent atom.

### Crystal data

**Table:**

C_16_H_12_ClFN_2_	*Z* = 2
*M**_r_* = 286.73	*F*(000) = 296
Triclinic, *P*1	*D*_x_ = 1.427 Mg m^−^^3^
Hall symbol: -P 1	Mo *K*α radiation, λ = 0.71073 Å
*a* = 7.3661 (8) Å	Cell parameters from 5222 reflections
*b* = 8.8967 (9) Å	θ = 2.5–31.0°
*c* = 11.5129 (12) Å	µ = 0.29 mm^−^^1^
α = 68.704 (1)°	*T* = 100 K
β = 74.468 (1)°	Plate, colourless
γ = 75.445 (1)°	0.55 × 0.50 × 0.25 mm
*V* = 667.25 (12) Å^3^	

### Data collection

**Table:**

Bruker APEXII CCD area-detector diffractometer	3930 independent reflections
Radiation source: fine-focus sealed tube	3633 reflections with *I* > 2σ(*I*)
graphite	*R*_int_ = 0.014
ω scans	θ_max_ = 31.4°, θ_min_ = 1.9°
Absorption correction: multi-scan (*SADABS*; Bruker, 2008)	*h* = −10→10
*T*_min_ = 0.858, *T*_max_ = 0.932	*k* = −12→12
8162 measured reflections	*l* = −16→16

### Refinement

**Table:**

Refinement on *F*^2^	Primary atom site location: structure-invariant direct methods
Least-squares matrix: full	Secondary atom site location: difference Fourier map
*R*[*F*^2^ > 2σ(*F*^2^)] = 0.034	Hydrogen site location: inferred from neighbouring sites
*wR*(*F*^2^) = 0.094	H-atom parameters constrained
*S* = 1.03	*w* = 1/[σ^2^(*F*_o_^2^) + (0.0533*P*)^2^ + 0.2404*P*] where *P* = (*F*_o_^2^ + 2*F*_c_^2^)/3
3930 reflections	(Δ/σ)_max_ = 0.001
181 parameters	Δρ_max_ = 0.49 e Å^−^^3^
0 restraints	Δρ_min_ = −0.30 e Å^−^^3^

### Special details

**Table:**

Geometry. All esds (except the esd in the dihedral angle between two l.s. planes) are estimated using the full covariance matrix. The cell esds are taken into account individually in the estimation of esds in distances, angles and torsion angles; correlations between esds in cell parameters are only used when they are defined by crystal symmetry. An approximate (isotropic) treatment of cell esds is used for estimating esds involving l.s. planes.
Refinement. Refinement of *F*^2^ against ALL reflections. The weighted *R*-factor *wR* and goodness of fit *S* are based on *F*^2^, conventional *R*-factors *R* are based on *F*, with *F* set to zero for negative *F*^2^. The threshold expression of *F*^2^ > 2σ(*F*^2^) is used only for calculating *R*-factors(gt) *etc*. and is not relevant to the choice of reflections for refinement. *R*-factors based on *F*^2^ are statistically about twice as large as those based on *F*, and *R*- factors based on ALL data will be even larger.

### Fractional atomic coordinates and isotropic or equivalent isotropic displacement parameters (Å^2^)

**Table:**

	*x*	*y*	*z*	*U*_iso_*/*U*_eq_	
Cl1	1.15120 (4)	−0.21591 (3)	0.79554 (2)	0.02245 (8)	
N1	1.24567 (12)	0.03932 (10)	0.60973 (8)	0.01634 (16)	
N2	0.55793 (12)	0.03799 (11)	0.74940 (8)	0.01744 (17)	
H18	0.4905	0.0291	0.7025	0.021*	
F1	0.24347 (10)	0.46529 (9)	1.02706 (7)	0.02663 (16)	
C7	1.09918 (14)	−0.03020 (11)	0.67819 (9)	0.01494 (17)	
C1	1.02460 (14)	0.25805 (11)	0.49326 (9)	0.01455 (17)	
C10	0.74539 (14)	−0.05999 (12)	0.75411 (9)	0.01625 (18)	
H10A	0.7513	−0.1557	0.7308	0.020*	
H10B	0.7657	−0.0977	0.8407	0.020*	
C8	0.90473 (13)	0.03112 (11)	0.66692 (9)	0.01406 (17)	
C5	1.36831 (15)	0.26375 (13)	0.44050 (10)	0.01949 (19)	
H5	1.4913	0.2165	0.4552	0.023*	
C6	1.21153 (14)	0.18553 (12)	0.51570 (9)	0.01520 (17)	
C9	0.87191 (13)	0.17731 (12)	0.57294 (9)	0.01522 (17)	
H9	0.7474	0.2240	0.5615	0.018*	
C14	0.21346 (15)	0.33659 (13)	0.88812 (10)	0.0206 (2)	
H14	0.0879	0.3910	0.8882	0.025*	
C11	0.48346 (14)	0.14581 (12)	0.81816 (9)	0.01552 (18)	
C2	0.99836 (15)	0.40819 (12)	0.39406 (9)	0.01792 (19)	
H17	0.8764	0.4568	0.3777	0.022*	
C13	0.32377 (16)	0.36116 (12)	0.95720 (10)	0.01941 (19)	
C3	1.15214 (16)	0.48201 (13)	0.32212 (10)	0.01972 (19)	
H3	1.1338	0.5806	0.2572	0.024*	
C12	0.59034 (14)	0.17592 (12)	0.88891 (9)	0.01760 (18)	
H12	0.7166	0.1235	0.8889	0.021*	
C4	1.33828 (16)	0.40979 (13)	0.34565 (10)	0.0210 (2)	
H4	1.4413	0.4616	0.2965	0.025*	
C15	0.29365 (14)	0.22899 (13)	0.81854 (10)	0.01916 (19)	
H15	0.2208	0.2116	0.7714	0.023*	
C16	0.50987 (15)	0.28339 (13)	0.95926 (10)	0.01933 (19)	
H16	0.5809	0.3021	1.0068	0.023*	

### Atomic displacement parameters (Å^2^)

**Table:**

	*U*^11^	*U*^22^	*U*^33^	*U*^12^	*U*^13^	*U*^23^
Cl1	0.01931 (13)	0.01748 (12)	0.02433 (13)	−0.00220 (9)	−0.00775 (9)	0.00240 (9)
N1	0.0150 (4)	0.0161 (4)	0.0176 (4)	−0.0027 (3)	−0.0047 (3)	−0.0039 (3)
N2	0.0128 (4)	0.0214 (4)	0.0208 (4)	−0.0023 (3)	−0.0036 (3)	−0.0099 (3)
F1	0.0308 (4)	0.0241 (3)	0.0268 (3)	−0.0030 (3)	−0.0011 (3)	−0.0146 (3)
C7	0.0154 (4)	0.0129 (4)	0.0162 (4)	−0.0015 (3)	−0.0049 (3)	−0.0037 (3)
C1	0.0149 (4)	0.0143 (4)	0.0149 (4)	−0.0024 (3)	−0.0030 (3)	−0.0052 (3)
C10	0.0139 (4)	0.0152 (4)	0.0183 (4)	−0.0028 (3)	−0.0019 (3)	−0.0045 (3)
C8	0.0137 (4)	0.0142 (4)	0.0148 (4)	−0.0028 (3)	−0.0028 (3)	−0.0049 (3)
C5	0.0159 (4)	0.0216 (5)	0.0208 (5)	−0.0063 (4)	−0.0028 (3)	−0.0050 (4)
C6	0.0147 (4)	0.0157 (4)	0.0161 (4)	−0.0033 (3)	−0.0033 (3)	−0.0054 (3)
C9	0.0134 (4)	0.0156 (4)	0.0164 (4)	−0.0016 (3)	−0.0037 (3)	−0.0049 (3)
C14	0.0181 (4)	0.0211 (5)	0.0200 (5)	−0.0001 (4)	−0.0030 (4)	−0.0063 (4)
C11	0.0152 (4)	0.0152 (4)	0.0143 (4)	−0.0044 (3)	−0.0012 (3)	−0.0025 (3)
C2	0.0197 (4)	0.0159 (4)	0.0169 (4)	−0.0022 (3)	−0.0044 (3)	−0.0035 (3)
C13	0.0242 (5)	0.0161 (4)	0.0165 (4)	−0.0047 (4)	−0.0001 (4)	−0.0054 (3)
C3	0.0239 (5)	0.0166 (4)	0.0174 (4)	−0.0054 (4)	−0.0037 (4)	−0.0029 (3)
C12	0.0165 (4)	0.0180 (4)	0.0180 (4)	−0.0051 (3)	−0.0030 (3)	−0.0043 (3)
C4	0.0210 (5)	0.0212 (5)	0.0202 (5)	−0.0088 (4)	−0.0012 (4)	−0.0043 (4)
C15	0.0165 (4)	0.0220 (5)	0.0196 (4)	−0.0016 (4)	−0.0051 (3)	−0.0073 (4)
C16	0.0221 (5)	0.0199 (4)	0.0175 (4)	−0.0077 (4)	−0.0037 (4)	−0.0050 (4)

### Geometric parameters (Å, °)

**Table:**

Cl1—C7	1.7434 (10)	C5—H5	0.93
N1—C7	1.3016 (13)	C9—H9	0.93
N1—C6	1.3732 (12)	C14—C13	1.3792 (15)
N2—C11	1.3792 (12)	C14—C15	1.3878 (14)
N2—C10	1.4388 (13)	C14—H14	0.93
N2—H18	0.86	C11—C12	1.4011 (14)
F1—C13	1.3661 (12)	C11—C15	1.4077 (14)
C7—C8	1.4217 (13)	C2—C3	1.3700 (14)
C1—C9	1.4133 (13)	C2—H17	0.93
C1—C6	1.4149 (13)	C13—C16	1.3737 (15)
C1—C2	1.4164 (13)	C3—C4	1.4128 (15)
C10—C8	1.5161 (13)	C3—H3	0.93
C10—H10A	0.97	C12—C16	1.3936 (14)
C10—H10B	0.97	C12—H12	0.93
C8—C9	1.3714 (13)	C4—H4	0.93
C5—C4	1.3726 (15)	C15—H15	0.93
C5—C6	1.4136 (13)	C16—H16	0.93
			
C7—N1—C6	117.48 (8)	C13—C14—C15	118.66 (10)
C11—N2—C10	121.76 (8)	C13—C14—H14	120.7
C11—N2—H18	119.1	C15—C14—H14	120.7
C10—N2—H18	119.1	N2—C11—C12	122.23 (9)
N1—C7—C8	126.51 (9)	N2—C11—C15	119.59 (9)
N1—C7—Cl1	115.47 (7)	C12—C11—C15	118.18 (9)
C8—C7—Cl1	118.01 (7)	C3—C2—C1	120.15 (9)
C9—C1—C6	117.92 (9)	C3—C2—H17	119.9
C9—C1—C2	123.09 (9)	C1—C2—H17	119.9
C6—C1—C2	118.98 (9)	F1—C13—C16	119.13 (9)
N2—C10—C8	113.32 (8)	F1—C13—C14	118.49 (9)
N2—C10—H10A	108.9	C16—C13—C14	122.38 (10)
C8—C10—H10A	108.9	C2—C3—C4	120.64 (9)
N2—C10—H10B	108.9	C2—C3—H3	119.7
C8—C10—H10B	108.9	C4—C3—H3	119.7
H10A—C10—H10B	107.7	C16—C12—C11	120.86 (9)
C9—C8—C7	115.61 (8)	C16—C12—H12	119.6
C9—C8—C10	122.70 (8)	C11—C12—H12	119.6
C7—C8—C10	121.69 (8)	C5—C4—C3	120.52 (9)
C4—C5—C6	119.71 (9)	C5—C4—H4	119.7
C4—C5—H5	120.1	C3—C4—H4	119.7
C6—C5—H5	120.1	C14—C15—C11	121.05 (9)
N1—C6—C5	118.51 (9)	C14—C15—H15	119.5
N1—C6—C1	121.50 (9)	C11—C15—H15	119.5
C5—C6—C1	119.99 (9)	C13—C16—C12	118.87 (9)
C8—C9—C1	120.95 (9)	C13—C16—H16	120.6
C8—C9—H9	119.5	C12—C16—H16	120.6
C1—C9—H9	119.5		
			
C6—N1—C7—C8	−0.94 (15)	C6—C1—C9—C8	−1.63 (14)
C6—N1—C7—Cl1	179.70 (7)	C2—C1—C9—C8	179.30 (9)
C11—N2—C10—C8	−82.06 (11)	C10—N2—C11—C12	4.96 (14)
N1—C7—C8—C9	0.82 (15)	C10—N2—C11—C15	−174.83 (9)
Cl1—C7—C8—C9	−179.83 (7)	C9—C1—C2—C3	178.48 (9)
N1—C7—C8—C10	−178.91 (9)	C6—C1—C2—C3	−0.58 (15)
Cl1—C7—C8—C10	0.43 (13)	C15—C14—C13—F1	−179.04 (9)
N2—C10—C8—C9	−14.02 (13)	C15—C14—C13—C16	0.18 (16)
N2—C10—C8—C7	165.69 (9)	C1—C2—C3—C4	0.04 (16)
C7—N1—C6—C5	179.69 (9)	N2—C11—C12—C16	−178.83 (9)
C7—N1—C6—C1	−0.29 (14)	C15—C11—C12—C16	0.96 (14)
C4—C5—C6—N1	179.83 (9)	C6—C5—C4—C3	−0.37 (16)
C4—C5—C6—C1	−0.19 (15)	C2—C3—C4—C5	0.44 (16)
C9—C1—C6—N1	1.53 (14)	C13—C14—C15—C11	0.14 (16)
C2—C1—C6—N1	−179.36 (9)	N2—C11—C15—C14	179.10 (9)
C9—C1—C6—C5	−178.45 (9)	C12—C11—C15—C14	−0.70 (15)
C2—C1—C6—C5	0.65 (14)	F1—C13—C16—C12	179.30 (9)
C7—C8—C9—C1	0.55 (14)	C14—C13—C16—C12	0.08 (16)
C10—C8—C9—C1	−179.73 (8)	C11—C12—C16—C13	−0.67 (15)

### Hydrogen-bond geometry (Å, °)

**Table:**

Cg1 and Cg2 are the centroids of the C1–C6 and C11–C16 rings, respectively.

**Table:**

*D*—H···*A*	*D*—H	H···*A*	*D*···*A*	*D*—H···*A*
N2—H18···N1^i^	0.86	2.30	3.1353 (12)	165
C4—H4···Cg2^ii^	0.93	2.91	3.7494 (13)	151
C10—H10A···Cg1^iii^	0.97	2.62	3.5365 (12)	157
C10—H10B···Cg2^iv^	0.97	2.98	3.8083 (11)	145

Symmetry codes: (i) *x*−1, *y*, *z*; (ii) −*x*+2, −*y*+1, −*z*+1; (iii) −*x*+2, −*y*, −*z*+1; (iv) −*x*+1, −*y*, −*z*+2.
